# Adulteration of Drugs

**Published:** 1890-02-15

**Authors:** 


### THERAPEUTICS.

### ADULTERATION OF DRUGS.

The *American Druggist* for December last, contains an article of some interest, written by Mr. A. Robinson McIlvaine, chairman of the Committee on Adulteration National Wholesale Drug Association, entitled, "What is an Adulteration in Pharmacy?" Mixing water with alcohol, alcohol with sulphuric ether, sulphuric ether with chloroform, and selling each as full strength preparations, is typical adulteration, a intentional deception being practised. But according to American law, an article is deemed adulterated if, when sold under a name recognized in the pharmacopœia it differs from the standard of strength, quality, or purity laid down therein. The writer also complains that when a medicine has been introduced and become popular enough to warrant its inclusion as an efficinal preparation, the pharmacopœia gives directions as to making it, which are different from those of the original recipe. And this often results in the pharmacopœial preparation being useless. Notwithstanding the number of new medicines now daily brought before the profession, most of the old officinal preparations maintain their ground. But as in America, so in this country, medicines prepared according to the directions given in the pharmacopœia are not, and cannot be always of exactly the same quality and strength. Irrespective of intentional adulteration, of which we believe there is little, drugs used in composition differ in strength themselves, and very slight accidental variation of chemical process may produce a different result. Medical men are often disappointed in the action of a medicine, and this frequently depends on some unintentional adulteration, and the remedy is not evident, for their greatest care cannot always secure exactly similar results.

